# Effects of different feed additives on growth performance, carcass characteristics, and apparent total nutrient digestibility of finishing beef steers

**DOI:** 10.1093/tas/txag034

**Published:** 2026-03-24

**Authors:** Enzo A Fioruci, Vinícius N Gouvêa, Reinaldo F Cooke, Matthew R Beck

**Affiliations:** Texas A&M AgriLife Research, High Plains Research and Extension Center, 3211 Russell Long Blvd, Canyon, TX, 79015, United States; Texas A&M AgriLife Research, High Plains Research and Extension Center, 3211 Russell Long Blvd, Canyon, TX, 79015, United States; Department of Animal Science, Texas A&M University, 474 Olsen Blvd, College Station, TX, 77843, United States; Department of Animal Science, Texas A&M University, 474 Olsen Blvd, College Station, TX, 77843, United States; Department of Animal Science, Texas A&M University, 474 Olsen Blvd, College Station, TX, 77843, United States

**Keywords:** feedlot, ionophore, onion extract, orange peel bitters

## Abstract

The objective was to evaluate the effects of feed additives on intake, growth performance, apparent digestibility of nutrients, and ruminal fermentation characteristics of finishing beef cattle. In Experiment 1 (Exp. 1), fifty-six Angus-crossbred steers (initial body weight = 356 ± 26 kg) were assigned to a randomized complete block design with a 2 × 2 factorial arrangement of treatments, including (1) monensin sodium at 33 mg/kg dry matter (MON; [−] or [+]), and (2) a proprietary multi‐component plant extract feed additive at 10 g/steer/day (PE; [−] or [+]) containing orange peel bitters, onion extract, specific fatty acids, and soybean and corn oil. This resulted in four treatment groups: CON (MON−/PE−), MON (MON+/PE−), PE (MON−/PE+), or MON + PE (MON+/PE+). The 168-day study included a 24-day adaptation and 144 days on a 90:10 concentrate-to-roughage finishing diet. In Experiment 2 (Exp. 2), eight ruminally cannulated steers were used in a duplicated 4 × 4 Latin Square design to evaluate the same treatments used in Exp. 1. No MON × PE interactions or main effect of MON and PE were observed for body weight or dry matter intake (DMI) in Exp. 1 (*P* ≥ 0.16). Steers fed PE+ (PE and MON + PE) had a greater average daily gain (2.37 kg/steer/day) during the adaptation period compared to steers fed PE− (CON and MON; 2.07 kg/steer/day; *P* = 0.04). Consequently, feed efficiency (GF) during the adaptation period was 9% greater for steers fed PE+ (*P* < 0.01). A tendency of MON × PE interaction was detected for GF from day 72 to 168 (*P* = 0.09), and steers MON + PE tended to have greater GF compared to MON (*P* = 0.06) but not compared to CON or PE (*P* ≥ 0.20). In Exp. 2, feeding MON+ (MON and MON + PE) tended to decrease DMI compared to steers fed MON− (CON and PE; *P* = 0.06). A MON × PE interaction was detected for ruminal concentration of propionate, butyrate, and isovalerate (*P* ≤ 0.02). Steers fed MON + PE and CON had a greater concentration of propionate compared to MON and PE (*P* ≥ 0.04). The ruminal concentration of butyrate was greater for steers fed PE + compared to steers fed PE− (*P* ≤ 0.001). Steers fed PE + had a lower acetate: propionate ratio compared to steers fed PE− (*P* = 0.04). Ruminal concentration of ammonia nitrogen was less for steers fed PE+ (*P* < 0.01). In summary, the feed additives evaluated had minimal effect on DMI. Feeding PE and MON + PE enhances growth performance during the initial days on feed and improves ruminal fermentation characteristics.

## Teaser

The combination of an ionophore and a multi-component plant extract feed additive improved body weight gain and rumen fermentation characteristics in finishing beef cattle.

## Introduction

Feed represents one of the largest expenses in feedlot cattle operations (Anderson et al. 2026), therefore, strategies to improve the efficiency of converting feed into body weight gain have been extensively investigated to enhance the economic profitability of these systems over the past years (McGrath et al. 2018). The use of technologies such as growth-promoting implants and feed additives has consistently demonstrated benefits in improving feed efficiency and increasing the profitability of feedlot operations (Duffield et al. 2012; Maxwell et al. 2015). According to Samuelson et al. (2016) more than 95% of the feedlot consulting nutritionists in the USA include an ionophore in finishing diets, with monensin sodium being the primary one used. The effects of monensin on ruminal fermentation are well established and include selective inhibition of Gram-positive bacteria, leading to improved energy efficiency (Booth 1985; Yang and Russell 1993; Butaye et al. 2003; Russell and Houlihan 2003), reduced dry matter intake (DMI) and enhanced feed efficiency (Duffield et al. 2012; Ellis et al. 2012).

In contrast, plant-derived compounds, also known as plant extracts and essential oils, have been investigated as natural alternatives to ionophores for modulating rumen fermentation and improving animal performance. This interest is largely driven by growing public concern about antibiotic residues and antimicrobial resistance (Tassoul and Shaver 2009; Khiaosa-Ard and Zebeli 2013). Many plant extracts have been shown to modulate ruminal fermentation to some extent (Calsamiglia et al. 2007). Flavonoids and polyphenols in onion and orange peel specifically have demonstrated many positive antimicrobial and antioxidant effects on animal nutrition (Ahmed et al. 2021; Ju et al. 2024a; Lambo et al. 2024; Olagunju et al. 2024; Yang et al. 2025). Most commercial feed additives combine different plant extracts to achieve synergistic effects on ruminal fermentation, thereby enhancing growth performance and feed efficiency in cattle (Kim et al. 2019; Meschiatti et al. 2019; Nasir et al. 2025).

We hypothesized that a multi-component plant extract feed additive containing onion extract, orange peel, and specific fatty acids could serve as an alternative to, or act synergistically with, monensin in finishing beef cattle. The objective of this experiment was to evaluate the effects of these feed additives on growth performance, carcass characteristics, and apparent total tract nutrient digestibility of finishing beef steers.

## Materials and methods

These experiments were conducted at the joint Texas A&M AgriLife Research and USDA-ARS research feedlot at Bushland, TX. All the procedures involving animals were reviewed and approved by the West Texas A&M University Institutional Care and Use Committee (protocol #2023.01.004).

### Experiment 1: Growth performance and carcass characteristics

#### Animals, housing, and experimental procedures

A total of fifty-six (*n* = 56) steers [British × Continental crossbreed; initial body weight (BW) = 356 ± 26 kg] were sourced in a local cattle auction located 8 km from the research site and used in this experiment. Steers were transported to the research site 14 days (day −14) before the beginning of the experiment and housed in 8 soil-surfaced pens (12 m × 26 m; 7 steers/pen), each pen equipped with one automatic self-contained feeding unit (SmartFeed; C-Lock Inc., Rapid City, SD) that allows individual intake evaluation, and one water trough per pen (Omni 1, Ritchie Inc., Conrad, Iowa). From day −14 to day −1 (acclimatation phase), all steers had ad libitum access to ground wheat hay and water.

Before the beginning of the experiment, all steers were individually weighed for two consecutive days (unshrunk BW; day −1 and day 1) before the morning feeding. On day −1, steers were vaccinated against respiratory disease (Vista Once SQ, Merck Animal Health, Kenilworth, NJ; 2 mL subcutaneously/steer) and clostridiosis (Covexin 8; Merck Animal Health; 5 mL subcutaneously/steer), orally dewormed (Safe Guard, Merck Animal Health; 5 mg/kg of BW/steer), provided an injectable dewormer (Dectomax; Zoetis, Parsippany, NJ; 10 mg of doramectin/50 kg BW; subcutaneously), and received an extended-release implant of trenbolone acetate and estradiol (Revalor XS; Merck Animal Health; 6 coated and 4 uncoated pellets placed under the skin on the posterior aspect of the ear). On day 1, steers were blocked in two BW blocks based on initial BW (average of the two consecutive weights) and treatments were assigned to steers (14 steers/treatment) in a 2 × 2 factorial arrangement, consisting of (1) ionophore monensin sodium (Rumensin 90, Elanco Animal Health, Indianapolis, IN) at 33 mg/kg dry matter (MON; absent [−] or present [+]), and (2) a proprietary multi‐component plant extract (PE) feed additive at 10 g/steer/day (PE absent [−] or present [+]) containing a blend of orange peel bitters, onion extract, specific fatty acids, and soybean and corn oil (CattlActive RA, Pro Earth Animal Health, Aurora, SD). This resulted in four treatment groups: CON (MON−/PE), MON (MON+/PE−), PE (MON−/PE+), MON + PE (MON+/PE+). The feed additives were incorporated into the mineral and vitamin supplement, which was included at 5% of the dietary DM (Table 1). The doses of MON and PE were set according to each product’s label recommendations to ensure that both were provided at commercially relevant levels commonly used under feedlot conditions.

**Table 1:** Ingredients and analyzed chemical composition of the diets (%, dry matter [DM] basis; Exp. 1 and 2).

*Item*	Step1	Step2	Step3	Finishing
***Days in each diet (Exp. 1)***	8	8	8	144
***Ingredients***				
*** Corn stalks***	40.0	30.0	20.0	10.0
*** Steam-flaked corn***	29.2	39.9	49.4	59.0
*** Wet corn gluten feed***	20.0	20.0	20.0	20.0
*** Dry distillers’ grain plus solubles***	5.00	4.00	4.00	4.00
*** Urea***	0.30	0.40	0.50	0.50
*** Corn oil***	0.50	0.70	1.10	1.50
*** Mineral and vitamin premix^1^***	5.00	5.00	5.00	5.00
***Analyzed composition***				
*** DM, % as fed***	78.1	77.8	77.5	77.2
*** Crude protein***	13.3	13.4	13.8	14.1
*** Neutral detergent fiber***	38.3	32.1	25.4	19.2
*** Ether extract***	3.46	3.75	4.31	4.83
*** Total digestible nutrients^2^***	76.0	80.3	84.8	89.3
*** Net Energy of maintenance^2^***	1.85	1.99	2.13	2.27
*** Net energy of gain^2^***	1.20	1.32	1.45	1.57

1 Custom-made blend containing the feed additives. Containing (DM basis): 27.3610% calcium carbonate, 22.6%, dry distiller grains plus solubles, 20.7% magnesium sulfate, 17.3% monocalcium phosphate, 7.02% salt, 4.03% potassium chloride, 0.25% manganese sulfate, 0.14% vitamin E (500 IU/g), 0.1382% zinc oxide, 0.0798% copper sulfate, 0.043% sodium selenite, 0.0056% vitamin A (1,000,000 IU/g), 0.0015% cobalt carbonate, 0.0012% ethylenediamine dihydroiodide, 0.0011% vitamin D (500,000 IU/g).

2 Estimated using tabular values (NASEM 2016). The net energy of maintenance and gain for corn oil were 5.60 and 3.85 Mcal/kg DM, respectively, and the total digestible nutrients for corn oil of 177% (NRC, 1996).

The experiment consisted of a 168-day feeding period. From days 1 to 71 (Phase 1), steers were housed in 8 pens (7 steers/pen) and fed using automatic self-contained feeding units (SmartFeed; C-Lock Inc., Rapid City, SD) that allow individual intake evaluation (one feeding unit/pen). During Phase 1, animals were rotated across pens every 30 days to account for any potential pen variation (Colombo et al. 2022). During phase 2, from day 72 to 168, steers within each treatment were moved to 28 soil-surfaced pens (8 × 25 m; 2 steers/pen and 7 pens/treatment), and intake was determined on a pen basis using the amount of feed delivered and the weekly collection and quantification of refusals. Treatments were provided from day 1 to day 168. Steers were transitioned from a high roughage diet (starting with 40% roughage diet) to a high concentrate diet (Table 1) over the course of 24-days (adaptation period) using a step-up protocol that gradually decreased the roughage source (corn stalks) from 40% (Step-1), to 30% (Step-2), to 20% (Step-3; eight days in each step), and finally to 10% of DM basis (finishing diet), which was fed for 144 days. The basal finishing diet was formulated to meet the nutrient requirements of finishing cattle (NASEM 2016) and to contain 10% roughage and 90% concentrate (Table 1). Each treatment was mixed individually using a feed wagon (Rotomix 84-8, Roto-Mix, Dodge City, KS) and delivered as a total mixed ration (TMR) once a day at 0800 h. To prevent diet cross-contamination, CON treatment was mixed before PE, followed by MON, and MON + PE. The feed mixer was completely emptied between loads. During Phase 2, feed bunks were visually scored each day using a 4-point scoring system at 0700 and 1900 h (Lundy et al. 2015), and feed delivery was adjusted so that feed bunks contained trace amounts of feed at 0630 h each day (slick or crumbs). Feed calls were made based on two consecutive bunk scores (1900 and 0700 h), and any increase or decrease in the current day’s feed delivery was consistent across all treatments and bunk scores. Individual intake and average daily gain were collected during Phase 1 (day 1 to day 71) of the experiment, and the steer was considered the experimental unit. During Phase 2, the pen was considered the experimental unit.

#### Sampling and laboratory analyses

Throughout the experiment, samples of feed ingredients were collected weekly for DM determination (105°C/24 h) and diet DM adjustments. The diet DM was maintained at 70% throughout the experiment. Refusals were collected weekly, dried (105°/24 h), and used for DMI calculation. Feed ingredients and TMR samples were collected every other week and stored at −20°C for subsequent laboratory analysis.

Paired-day, unshrunk BW were collected on days −1 and 1 (at the beginning of the experiment), 23 and 24 (at the end of the adaptation period), 70 and 71 (end of Phase 1; individual data collection), 167 and 168 (end of the experiment). The average daily gain (ADG) was calculated using the initial and final BW, and feed efficiency (G: F) was calculated as the ratio of ADG to DMI. Steers were harvested at a commercial packing plant located approximately 1 h from the research site (Tyson Foods, Amarillo, TX) on day 168, and hot carcass weight (HCW) was collected. After 24 h of chilling, carcass data, including marbling score, USDA quality grade, 12th rib fat depth, Longissimus Muscle (LM) area, kidney, pelvic, and heart (KPH) fat, and calculated yield grade were collected by trained personnel from the Beef Carcass Research Center (West Texas A&M University, Canyon, TX). The dressing percentage was calculated by expressing HCW as a percentage of final BW.

***Statistical analysis***: Data were analyzed as a randomized complete block design in a 2 × 2 factorial arrangement of treatments (MON [− or +] and PE [− or +]) using the MIXED procedure of SAS (SAS Institute Inc., Cary, NC, USA, version 9.4). Steer (Phase 1) or pen (Phase 2) was the experimental unit. The statistical model in Phase 1 included the fixed effects of MON, PE, and the resulting interaction between MON × PE. The initial BW block and steer(treatment) were included as a random effect. The statistical model in Phase 2 included the fixed effect of MON, PE, and the resulting interaction between MON × PE. The BW block and pen(treatment) were included as a random effect. The Kenward-Roger approximation method was used to determine the denominator degrees of freedom for testing fixed effects. Marbling score, quality grade, and liver score were analyzed using a binomial distribution by the GLIMMIX procedure of SAS with the same fixed and random effects described previously. The link function was logit, and the degrees of freedom were adjusted using the Kenward–Roger method. All results were reported as least-square means. Differences among treatments were considered significant when *P* ≤ 0.05, with trends noted when 0.05 < *P* ≤ 0.10.

### Experiment 2: nutrient digestibility and ruminal fermentation characteristics

#### Animals, housing, and experimental procedures

Eight ruminally cannulated steers [British and British × Continental; average BW = 403 ± 43.5 lb.] were used in a duplicated 4 × 4 Latin Square design to evaluate the effects of the same treatments used in Exp. 1 on dry matter intake, nutrient digestibility, rumination time, and rumen fermentation characteristics. Before the beginning of the experiment, steers were individually weighed and vaccinated against respiratory disease (Vista Once SQ, Merck Animal Health, Kenilworth, NJ; 2 mL subcutaneously/steer) and clostridiosis (Covexin 8, Merck Animal Health; 5 mL subcutaneously/steer), dewormed orally (SafeGuard, Merck Animal Health; 5 mg of fenbendazole/kg of BW/steer), and administered pour-on insecticide (CyLence, Bayer Animal Health, Shawnee Mission, KS; 8 mL/steer). No growth implant was administered. Steers were housed in eight individual pens (5 × 5 m) with ad libitum access to fresh water (OmniFount 2; Ritchie Industries Inc., Conrad, IA) during the entire experiment.

Treatments were the same as described in Exp. 1. The finishing diet was offered as a TMR once daily at 0800 h. Diets were mixed twice a week (Monday and Friday) in the morning, using a feed wagon (Rotomix 84-8, Roto-Mix, Dodge City, KS), unloaded into plastic feed bins, and stored in a walk-in cooler (5°C) for up to 3 days. The TMR was weighed daily into plastic buckets using a platform scale with 5 g readability (Ohaus SD Series; Ohaus Corp., Parsippany, NJ) and manually delivered to each steer once a day. Feed bunks were visually scored each day by estimating the amount of feed remaining at 1800 and 0600 h, with a numeric score assigned at each viewing according to Lundy et al. (2015), and a target score of 1 at 0600 h to ensure ad libitum DMI.

#### Experimental periods and sampling

Experimental periods (*n* = 4) were 16-d in length, consisting of 10 days of diet adaptation, and 6 days of collection. Steers were fitted with a collar with sensors (AllFlex Livestock Intelligence, Madison, WI) to continuously record rumination time throughout the trial (Lockard et al. 2021). Rumination time (min/h) was recorded in 2-h intervals, and data from days 11 to 15 (sampling days) were used to estimate rumination behaviors. Ruminal pH was also monitored continuously throughout the study using submersible pH probes (SmaxTec, Graz, Austria), which were manually inserted into the rumen before the start of the experiment and programmed to record pH at 10-minute intervals (Ceconi et al. 2022).

The intake and the apparent digestibility of nutrients (dry matter, crude protein, neutral detergent fiber, and ether extract) were evaluated from day 11 through day 15. Chromium oxide was used as an indigestible marker for estimating fecal output. Chromium oxide was weighed at 7.5 grams/dose into #07 large animal gelatin capsules (Torpac Inc., Fairfield, New Jersey) and dosed intraruminally immediately before the morning feeding at 0800 and again at 1800 h daily from day 3 through day 15. Fecal grab samples were collected manually from the rectum of each steer twice daily at 0800 and again at 1800 h from day 11 to day 15 (Benton et al. 2015). Fecal samples were then frozen at −20°C for later laboratory analyses. Samples of diets, feed ingredients, and refusals were collected daily during the sampling days and frozen at −20°C for subsequent analyses. Refusals were collected daily before the morning feeding. A subsample was dried (105°C/24 h) for DMI calculation, and another subsample was stored at −20°C for subsequent laboratory analysis as described below.

On day 16, approximately 25 mL of ruminal fluid was manually obtained from each steer via the ruminal cannula at 0, 4, 8, and 12 h after feeding. Ruminal fluid samples were squeezed through 4 layers of cheesecloth into two 50 mL containers, one for analysis of volatile fatty acids (VFA; Palmquist and Conrad 1971) and one for ammonia nitrogen (NH_3_-N; Chaney and Marbach 1962). The samples were placed in a cooler with dry ice immediately after sampling. The VFA samples were not acidified. Samples for NH_3_-N were acidified using 1% H_2_SO_4_ (21 mL rumen fluid and 3 mL acid). Samples were inverted three times to mix and stored at −20°C until analysis.

## Laboratory analysis and calculations

At the end of both experiments, samples of diets, feed ingredients, feed refusals (Exp. 1 and 2), and fecal samples (Exp. 2) were thawed, composited (by animal and period in Exp. 2), dried in a forced-air oven at 55°C for 72 h, and ground through a 1-mm screen of a Willey-type mill (Thomas Model 4 Wiley Mill, Thomas Scientific, Swedesboro, New Jersey). Samples were analyzed for DM at 105°C (method 930.15; (AOAC—Official methods of analysis, 2000), neutral detergent fiber (NDF) according to Van Soest et al. (1991) using sodium sulfite and heat-stable α-amylase, and acid detergent fiber (ADF) modified for an Ankom 200 fiber analyzer (Ankom Technology Corp., Macedon, NY), ether extract (EE) using an ANKOM XT15 Extractor (Ankom Technologies; Komarek et al. 2019), and CP by combustion using a VarioMax Cube (Elementar Americas Inc., Ronkonkoma, NY; method 990.03; AOAC 2000).

Chromium concentration in fecal samples was analyzed by atomic absorption using an air-acetylene flame (Model 351 AA/AE Spectrophotometer, Instrumentation Laboratory, Inc., Lexington, MA).

Minimum, mean, and maximum pH values were determined, and the time that ruminal pH remained below 5.2 and the area under the curve were calculated as described by Ceconi et al. (2022). Daily rumination was calculated as described by Lockard et al. (2021) by summing daily 2-h intervals and averaging across 24-h periods. Based on observed DMI and NDF intakes, rumination was expressed in min/kg of DMI and min/kg of NDF intake.

Ruminal samples were prepared for analysis by thawing, centrifuging (15,000 × *g* for 10 min at room temperature for VFA and NH_3_–N; Cappellozza et al. 2013), and collecting the supernatant. The VFA concentration was analyzed by gas chromatography with a flame ionization detector and a fused-silica capillary column of 25 m in length and 320 μM internal diameter (Agilent 7890A, Agilent, Santa Clara, CA) as described by (Polizel et al. 2021). The NH_3_-N concentration was determined using a colorimetric method as described by Chaney and Marbach (1962), adapted for a microplate analysis with absorbance measured at 550 nm using a microplate reader (Synergy H1, Agilent) as described by Antunes et al. (2025).

***Statistical Analysis:*** Data were analyzed as a duplicated 4 × 4 Latin Square (LSQ) design with a 2 × 2 factorial arrangement of treatments (MON [− or +] and PE [− or +]) using the MIXED procedure of SAS (SAS Institute Inc., Cary, NC, USA, version 9.4). The statistical model for intake, apparent total nutrient digestibility, and rumination included the fixed effect of MON, PE, and the resulting interaction between MON × PE. The LSQ, period(LSQ), and steer(LSQ) were considered random effects (Kaps and Lamberson 2009). The NH_3_-N and VFA concentrations were analyzed as repeated measurements, and the statistical model included the effect of MON, PE, time, and the resulting interactions between MON × PE and MON × PE×time. Time was the repeated measure, and the subject was steer (treatment). Because this last interaction was not significant for any of the response variables analyzed, time was excluded from the model, and treatment means were reported to simplify data interpretation (Antunes et al. 2025). The Kenward-Roger approximation method was used to determine the denominator degrees of freedom for testing fixed effects. All results were reported as least-square means. Differences among treatments were considered different when *P* ≤ 0.05, with trends noted when 0.05 < *P* ≤ 0.10.

## Results

In Exp. 1, no MON × PE interactions or main effect of MON and PE were observed for BW or DMI (*P* ≥ 0.16; Table 2). Steers fed PE+ (PE and MON + PE) had greater ADG (2.37 kg/steer/day) during the adaptation period (Phase 1; days 1–24) compared to steers fed PE- (CON and MON; 2.07 kg/steer/day; main effect of PE; *P* = 0.04; Table 2). No other effects were observed on ADG (*P* ≥ 0.11; Table 2). Consequently, G: F during the adaptation period (Phase 1; days 1–24) was 9% greater for steers fed PE+ (PE and MON + PE) compared to steers fed PE- (CON and MON; main effect of PE; *P* < 0.01). A MON × PE interaction was detected for G: F from days 25 to 71 (Phase 1; *P* = 0.02), and steers fed MON had greater G: F compared to MON + PE and CON (*P* ≤ 0.05) but not compared to PE (*P* = 0.36; Table 2). A tendency of MON × PE interaction was detected for G: F from day 72 to 168 (Phase 2; *P* = 0.09; Table 2), and steers MON + PE tended to have greater G: F compared to MON (*P* = 0.06) but not compared to CON or PE (*P* ≥ 0.20). No other differences in G: F were observed between treatments (*P* ≥ 0.42; Table 2). No effects of MON, PE, or MON × PE interaction were observed for any of the carcass characteristics evaluated (*P* ≥ 0.12; Table 3).

**Table 2:** Effect of feed additives (ionophore monensin sodium [MON] or a proprietary multi‐component plant extract [PE]) on growth performance of finishing beef steers (Exp. 1).

*Item*	Treatments^1^				SEM^2^	*P*-value		
	CON (MON−/PE−)	MON (MON+/PE−)	PE (MON−/PE+)	MON + PE (MON+/PE+)		MON	PE	MON × PE
***Body weight, kg***								
*** Day 1***	356	357	357	355	21.2	0.88	0.89	0.83
*** Day 24***	408	405	414	413	23.9	0.66	0.27	0.86
*** Day 71***	489	492	493	494	19.5	0.75	0.71	0.88
*** Day 168***	666	668	672	672	11.2	0.77	0.97	0.95
***Dry matter intake, kg***								
***Phase 1***								
*** Days 1 to 24***	9.44	8.88	8.92	8.10	0.63	0.26	0.28	0.83
*** Days 25 to 71***	12.0	11.5	11.1	11.9	0.46	0.74	0.55	0.16
*** Days 1 to 71***	11.0	10.6	10.3	10.7	0.50	0.98	0.52	0.37
***Phase 2***								
*** Days 72 to 168***	12.7	12.6	13.1	12.4	0.40	0.82	0.28	0.47
***Overall***								
*** Days 1 to 168***	11.9	11.7	11.7	11.7	0.32	0.83	0.77	0.76
***Average daily gain, kg***								
***Phase 1***								
*** Days 1 to 24***	2.14	2.00	2.40	2.33	0.16	0.48	0.04	0.80
*** Days 25 to 71***	1.69	1.86	1.70	1.62	0.094	0.57	0.15	0.11
*** Days 1 to 71***	1.87	1.91	1.92	1.89	0.07	0.91	0.80	0.62
***Phase 2***								
*** Days 72 to 168***	1.86	1.81	1.83	1.87	0.10	0.83	0.98	0.65
***Overall***								
*** Days 1 to 168***	1.84	1.85	1.87	1.88	0.07	0.64	0.81	0.99
***Gain: feed ratio***								
***Phase 1***								
*** Days 1 to 24***	0.241	0.226	0.275	0.299	0.025	0.81	<0.01	0.29
*** Days 25 to 71***	0.144^b^	0.166^a^	0.156^ab^	0.138^b^	0.011	0.80	0.31	0.02
*** Days 1 to 71***	0.175	0.182	0.188	0.181	0.008	0.97	0.42	0.38
***Phase 2***								
*** Days 72 to 168***	0.146^xy^	0.133^y^	0.140^xy^	0.153^x^	0.007	0.98	0.32	0.09
***Overall***								
*** Days 1 to 168***	0.156	0.158	0.159	0.162	0.005	0.58	0.66	0.87

1 Control (MON−/PE−) = no feed additive; MON (MON+/PE−) = ionophore monensin sodium at 33 mg kg dry matter (Rumensin 90, Elanco Animal Health, Indianapolis, IN, USA); PE (MON−/PE+) = proprietary multi‐component plant extract at 10 g/steer/day (CattlActive RA, Pro Earth Animal Health, Aurora, SD, USA); MON + PE (MON+/PE+) = combination of ionophore monensin sodium (Rumensin 90, Elanco Animal Health) and proprietary multi‐component plant extract (CattlActive RA, Pro Earth Animal Health).

2 Pooled standard error of the mean.

ab Means that do not have common superscript letters are different (*P* ≤ 0.05).

xy Means that do not have common superscript letters tend to differ (0.10 ≥ *P* > 0.05).

**Table 3:** Effect of feed additives (ionophore monensin sodium [MON] or a proprietary multi‐component plant extract [PE]) on carcass characteristics of finishing beef steers (Exp. 1).

*Item*	Treatments^1^				SEM^2^	*P*-value		
	CON (MON−/PE−)	MON (MON+/PE−)	PE (MON−/PE+)	MON + PE (MON+/PE+)		MON	PE	MON × PE
***Carcass characteristics***								
*** Hot carcass weight, kg***	423	424	429	426	14.5	0.98	0.66	0.79
*** Dressing, %***	63.6	62.9	63.9	63.4	0.43	0.17	0.33	0.86
*** Ribeye area, cm^2^***	103	98.6	103	103	3.01	0.45	0.44	0.55
*** Fat thickness, cm***	1.63	1.63	1.57	1.44	0.15	0.66	0.41	0.66
*** Yield grade***	3.38	3.22	2.97	2.95	0.239	0.62	0.12	0.86
*** Color score***	5.00	5.00	5.15	5.00	0.089	0.40	0.40	0.40
***Quality grade, %, (n/n)***								
*** Prime***	− (1/14)	− (0/14)	− (0/13)	− (1/14)	–	–	–	–
*** Premium Choice***	14.2 (2/14)	21.3 (3/14)	30.6 (4/13)	21.3 (3/14)	11.3	0.99	0.48	0.48
*** Choice***	64.4 (9/14)	42.8 (6/14)	69.3 (9/13)	50.0 (7/14)	13.6	0.15	0.66	0.96
*** Select***	− (2/14)	− (5/14)	− (0/13)	− (3/14)	–	–	–	–
***Edible livers, %***	50.0	78.7	62.0	71.5	14.1	0.16	0.94	0.49

1 Control (MON−/PE−) = no feed additive; MON (MON+/PE−) = ionophore monensin sodium at 33 mg kg dry matter (Rumensin 90, Elanco Animal Health, Indianapolis, IN, USA); PE (MON−/PE+) = proprietary multi‐component plant extract at 10 g/steer/day (CattlActive RA, Pro Earth Animal Health, Aurora, SD, USA); MON + PE (MON+/PE+) = combination of ionophore monensin sodium (Rumensin 90, Elanco Animal Health) and proprietary multi‐component plant extract (CattlActive RA, Pro Earth Animal Health).

2 Pooled standard error of the mean.

In Exp. 2, feeding MON+ (MON and MON + PE) tended to decrease DMI compared to steers fed MON- (CON and PE; main effect of MON; *P* = 0.06; Table 4), but no other effects were observed on nutrient intake or apparent total tract digestibility of nutrients (*P* ≥ 0.18). Treatments did not affect ruminal pH (*P* ≥ 0.11; Table 5), although the numerical decrease in the AUC for pH 5.2 for steers fed MON + PE compared to CON (*P* > 0.05; Table 5). A MON × PE interaction was detected for total ruminal VFA concentration (*P* = 0.04; Table 5). Steers fed PE had less VFA concentration compared to CON (*P* = 0.02) but not compared to MON or MON + PE (*P* ≥ 0.10).

**Table 4:** Effect of feed additives (ionophore monensin sodium [MON] or a proprietary multi‐component plant extract [PE]) on the apparent total tract digestibility of finishing beef steers (Exp.2).

*Item*	Treatments^1^				SEM^2^	*P*-value		
	CON (MON–/PE–)	MON (MON+/PE–)	PE (MON–/PE+)	MON + PE (MON+/PE+)		MON	PE	MON × PE
***Intake, kg/steer/day***								
*** Dry matter***	12.6	11.6	12.5	11.9	0.60	0.06	0.86	0.63
*** Crude protein***	1.60	1.54	1.56	1.47	0.137	0.56	0.66	0.91
*** Ether extract***	0.428	0.426	0.461	0.411	0.048	0.32	0.74	0.37
*** Neutral detergent fiber***	2.93	2.75	2.99	2.68	0.203	0.22	0.97	0.72
***Apparent total tract digestibility, %***								
*** Dry matter***	77.3	77.2	77.6	76.2	1.29	0.50	0.78	0.58
*** Crude protein***	70.3	70.8	71.3	69.5	2.67	0.81	0.95	0.66
*** Ether extract***	86.7	88.9	87.4	87.0	1.31	0.38	0.58	0.21
*** Neutral detergent fiber***	44.6	49.1	46.2	41.6	3.77	0.99	0.38	0.18

1 Control (MON–/PE–) = no feed additive; MON (MON+/PE-) = ionophore monensin sodium at 33 mg kg dry matter (Rumensin 90, Elanco Animal Health, Indianapolis, IN, USA); PE (MON-/PE+) = proprietary multi‐component plant extract at 10 g/steer/day (CattlActive RA, Pro Earth Animal Health, Aurora, SD, USA); MON + PE (MON+/PE+) = combination of ionophore monensin sodium (Rumensin 90, Elanco Animal Health) and proprietary multi‐component plant extract (CattlActive RA, Pro Earth Animal Health).

2 Pooled standard error of the mean.

**Table 5:** Effect of feed additives (ionophore monensin sodium [MON] or a proprietary multi‐component plant extract [PE]) on ruminal pH, volatile fatty acids (VFA), and ammonia nitrogen (NH_3_-N) of finishing beef steers (Exp.2).

*Item*	Treatments^1^					*P*-value		
	CON (MON–/PE–)	MON (MON+/PE–)	PE (MON–/PE+)	MON + PE (MON+/PE+)	SEM^2^	MON	PE	MON × PE
***Ruminal pH***								
*** Maximum pH***	6.18	6.07	6.17	6.10	0.081	0.11	0.85	0.79
*** Minimum pH***	4.71	4.75	4.72	4.73	0.078	0.37	0.80	0.44
*** Average pH***	5.22	5.23	5.27	5.24	0.070	0.52	0.13	0.36
*** Time below 5.2, hours/d***	0.498	0.493	0.505	0.512	0.120	0.99	0.70	0.87
*** Area under the curve pH 5.2***	1006	979	879	759	221	0.11	0.11	0.96
***Total VFA concentration, mM***	199^a^	189^ab^	179^b^	193^ab^	12.3	0.76	0.19	0.04
***VFA proportion, mol/100 mol***								
*** Acetate***	45.8	44.2	43.3	43.1	1.13	0.36	<0.05	0.25
*** Propionate***	28.0^a^	26.9^b^	27.1^b^	28.5^a^	1.17	0.58	0.35	<0.01
*** Butyrate***	14.0^b^	15.4^b^	17.8^a^	16.8^a^	1.55	0.66	<0.01	0.02
*** Valerate***	6.73	6.84	6.10	6.53	0.748	0.29	0.08	0.53
*** Isobutyrate***	1.93	2.00	1.88	1.92	0.109	0.42	0.35	0.82
*** Isovalerate***	3.73^bc^	4.69^a^	4.44^ab^	2.97^c^	0.77	0.38	0.08	<0.01
*** Acetate: propionate ratio***	1.67	1.70	1.64	1.56	0.094	0.24	0.04	0.23
*** Ruminal N-NH_3_, mg/dL***	18.2	20.0	15.8	15.1	1.51	0.47	<0.01	0.11

1 Control (MON–/PE–) = no feed additive; MON (MON+/PE–) = ionophore monensin sodium at 33 mg kg dry matter (Rumensin 90, Elanco Animal Health, Indianapolis, IN, USA); PE (MON–/PE+) = proprietary multi‐component plant extract at 10 g/steer/day (CattlActive RA, Pro Earth Animal Health, Aurora, SD, USA); MON + PE (MON+/PE+) = combination of ionophore monensin sodium (Rumensin 90, Elanco Animal Health) and proprietary multi‐component plant extract (CattlActive RA, Pro Earth Animal Health).

2 Pooled standard error of the mean.

ab Means that do not have common superscript letters are different (*P* < 0.05).

Steers fed PE+ (PE and MON + PE) had decreased ruminal concentration (mol/100 mol) of acetate compared to steers fed PE- (MON and CON; main effect of PE; *P* < 0.05; Table 5). A MON × PE interaction was detected for ruminal concentration of propionate, butyrate, and isovalerate (*P* ≤ 0.02; Table 5). Steers fed MON + PE and CON had a greater concentration of propionate compared to MON and PE (*P* ≥ 0.04). The ruminal concentration of butyrate was greater for steers fed PE and MON + PE compared to CON and MON (*P* ≤ 0.001). Ruminal concentration of isovalerate was greater for steers fed MON compared to CON and MON + PE (*P* ≤ 0.001) but not compared to PE (*P* = 0.54; Table 5).

Steers fed PE+ (PE and MON + PE) had a lower acetate: propionate ratio compared to steers fed PE- (CON and MON; main effect of PE; *P* = 0.04; Table 5). Ruminal concentration of NH_3_-N was also less for steers fed PE+ (PE and MON + PE) compared to steers fed PE- (CON and MON; main effect of PE; *P* < 0.01; Table 5). Time spent ruminating (min/day or min/kg DMI) was greater for PE+ (PE and MON + PE) compared to steers fed PE- (CON and MON; main effect of PE; *P* ≤ 0.04; Table 6).

**Table 6:** Effect of feed additives (ionophore monensin sodium [MON] or a proprietary multi‐component plant extract [PE]) on rumination time of finishing beef steers (Exp. 2).

*Item*	Treatments^1^					*P*-value		
	CON (MON–/PE–)	MON (MON+/PE–)	PE (MON–/PE+)	MON + PE (MON+/PE+)	SEM^2^	MON	PE	MON × PE
***Minutes/day***	383	362	414	405	36.8	0.40	0.04	0.73
***Minutes/kg of DMI***	30.3	31.9	33.3	34.7	2.63	0.22	0.03	0.89
***Minutes/kg of NDF intake***	129	144	140	156	13.2	0.17	0.31	0.94

DMI = dry matter intake; NDF = neutral detergent fiber.

1 Control (MON–/PE–) = no feed additive; MON (MON+/PE–) = ionophore monensin sodium at 33 mg kg dry matter (Rumensin 90, Elanco Animal Health, Indianapolis, IN, USA); PE (MON–/PE+) = proprietary multi‐component plant extract at 10 g/steer/day (CattlActive RA, Pro Earth Animal Health, Aurora, SD, USA); MON + PE (MON+/PE+) = combination of ionophore monensin sodium (Rumensin 90, Elanco Animal Health) and proprietary multi‐component plant extract (CattlActive RA, Pro Earth Animal Health).

2 Pooled standard error of the mean.

ab Means that do not have common superscript letters are different (*P* < 0.05).

## Discussion

Monensin is an ionophore known to enhance the efficiency of energy metabolism in ruminants, which ultimately leads to a reduction in DMI (Butaye et al. 2003; Page 2003). In a meta-analysis on the effects of feeding monensin to beef cattle, Duffield et al. (2012) reported that supplementing growing and finishing cattle with monensin at an average concentration of 28.1 mg/kg of feed (DM basis) reduced DMI by 3.1%, increased ADG by 2.5%, and improved G: F by 6.4%. Duffield et al. (2012) also observed a decline in the positive effects of feeding monensin over time, with improvements G: F decreasing from 8% in the 1970s to 3.5% after 2000. According to these last authors, this reduction may be attributed to changes in management practices and production systems. Many recent studies reported no significant effect of monensin on DMI or GF of beef cattle (Boardman et al. 2020; Gouvêa et al. 2021), although monensin is still the primary ionophore included in feedlot diets (Samuelson et al. 2016). Similarly, no differences in DMI were observed between treatments in Exp. 1, and a tendency for reduced DMI on steers fed MON was observed in Exp. 2. It is possible that the good feeding management practices employed during the research project, such as consistent feeding time and consistent bunk management, can reduce the likelihood of monensin-driven changes in feeding behavior, such as decreasing meal size and increasing meal events (Gibb et al. 2001).

As an alternative to ionophores, various plant extracts have been evaluated for their ability to enhance ruminal fermentation in ruminants over the past few years (Calsamiglia et al. 2007; Vasta et al. 2019; Abd’quadri-Abojukoro and Nsahlai 2023). Interest in ionophore alternatives is largely of interest due to increasing restrictions on the use of antibiotics in livestock production (Tassoul and Shaver 2009; Khiaosa-Ard and Zebeli 2013; Matheou et al. 2025). However, many commercial products combine various plant extracts to produce synergistic effects on ruminal fermentation, potentially improving growth performance and feed efficiency of cattle (Meschiatti et al. 2019; Kim et al. 2019; Nasir et al. 2025), which complicates the ability to determine the individual contribution of each compound to ruminal fermentation characteristics and growth performance. Among plant bioactive compounds, polyphenols, commonly found in citrus fruits, have been recognized for their beneficial antioxidant effects on human (Williamson 2017; Mas-Capdevila et al. 2020) and animal health (Ju et al. 2024), including positive modulation of ruminal fermentation. Onion extract also has a high concentration of polyphenols and flavonoids, which have demonstrated antioxidant and antimicrobial activity (Ye et al. 2013).

According to Amiri et al. (2019), supplementing onion extract to suckling lambs increased daily weight gain compared to a control diet without additives, which was attributed to higher DMI, likely driven by increased plasma ghrelin and decreased leptin concentrations. In the current experiment, no differences in DMI were observed between treatments. The increase in ADG and GF during the adaptation period (days 1–24) observed in Exp. 1 for steers fed PE and PE + MON is probably attributable to shifts in ruminal fermentation characteristics, such as reduced acetate: propionate ratio, which is confirmed by results in Exp. 2. The improved ruminal fermentation characteristics, such as decreased acetate and acetate: propionate ratio, and increased butyrate, along with decreased ruminal N-NH_3_ can support the increase in growth performance observed during the initial days on fed in steers fed PE or MON + PE in the current study.

The broad-spectrum antimicrobial activity of onion extract observed in vitro by Ye et al. (2013) indicates its potential to affect rumen microbiota. Such modulation could alter fermentation characteristics, including VFA production, as observed in the current study, which are key factors in cattle growth performance. According to Ye et al. (2013), onion extract had a potent inhibitory effect against gram-positive bacteria. Since cellulolytic bacteria are mainly gram-positive bacteria (Alam et al. 2024), their inhibition could reduce cellulose degradation and, consequently, decrease acetate production in the rumen as observed in the current study (Stewart et al. 1997; Hua et al. 2022). This would be expected to decrease acetate: propionate ratio as observed in the current study, without necessarily affecting DMI. It is important to highlight that no differences in NDF digestibility were detected in the current study.

Like the current study, Ahmed et al. (2021) observed that a mixture of garlic and citrus extract increased the in vitro concentration of butyrate, which is known to stimulate papillae growth (Liu et al. 2019), increasing the surface area for total VFA absorption and potentially increasing energy uptake and growth. Recently, Zhong et al (2023) observed that dietary supplementation with butyrate improved growth and gastro-intestinal development in pre-weaned dairy bulls by inhibiting inflammation and improving nutrient metabolism. Although rumen papillae and intestinal health markers were not evaluated in the current study, the performance findings associated with the increases in ruminal butyrate concentration may enhance nutrient absorption and growth in finishing cattle by promoting gastrointestinal development and improving energy efficiency. According to Yu et al. (2024), citrus-derived flavonoids have a direct inhibitory effect on *Entodinium* protozoa, which was directly responsible for lower ruminal ammonia concentration. Ruminal protozoa produce oligopeptides and amino acids during the digestion of engulfed microorganisms, and these metabolites subsequently serve as substrates for amino-acid-fermenting bacteria, as descried by Williams and Coleman (1992).

Dietary supplementation with orange peel has been shown to increase BW and improve feed conversion in broilers (Ebrahimi et al. 2013; Abd El Latif et al. 2023), effects that were primarily attributed to its antioxidant activity, as indicated by increased blood superoxide dismutase concentrations. Although antioxidant activity was not evaluated in the current study, it is possible that the antioxidant effect can benefit growth in ruminants, as described by (Celi and Gabai, 2015). In agreement with the current study, Ahmed et al. (2021) also reported an increase in in vitro butyrate concentration when orange peel was added to the diets. Ju et al. (2024) supplemented dairy cows during the transition period with orange peel (4 g/cow/day) and observed increases in milk yield, milk protein, and lactose content, which were accompanied by higher total VFA concentrations, particularly propionate and butyrate.

Increased rumination time is often associated with improved rumen function and fermentation stability. Longer rumination enhances saliva production, which buffers rumen pH and prevents prolonged periods of suboptimal acidity (Castillo-Lopez et al. 2014). In the current study, steers fed PE spent more time ruminating, which likely contributed to the numerically lower area under the curve for pH 5.2, indicating fewer periods of ruminal acidosis. The specific mechanism by which specific plant extracts influence rumination time is not fully elucidated; however, citrus-derived flavonoids have been shown to exert a direct inhibitory effect on *Entodinium* protozoa (Yu et al. 2024). This reduction in protozoal activity can slow the overall fermentation rate and increase ruminal retention time, thereby allowing the fiber mat to remain more intact and promoting longer rumination. Additionally, the fermentation profile showed decreased acetate and increased butyrate concentrations. Butyrate, in particular, stimulates rumen epithelial development and papillae growth, further supporting efficient absorption of VFA and overall rumen health. Together, these changes suggest that increased rumination improved fermentation efficiency and stabilized rumen conditions, which can enhance energy utilization and potentially growth performance.

In summary, the feed additives evaluated in the current study had minimal effect on DMI. Feeding PE and MON + PE improves growth performance during the initial days on feed and enhances ruminal fermentation characteristics in beef cattle fed finishing feedlot diets.

## List of abbreviations

ADG: Average Daily Gain
C2: C3: Acetate to Propionate ratio
BW: Body Weight
CA: CattlActive
CON: Control
CP: Crude Protein
DMI: Dry Matter Intake
DM: Dry Matter
EE: Ether Extract
GF: Gain to Feed ratio (Feed Efficiency)
HCW: Hot Carcass Weight
ION: Ionophore
KPH: Kidney, Pelvic, and Heart fat
LSQ: Latin Square design
MON: Monensin
MON+CA: Monensin + CattlActive
NDF: Neutral Detergent Fiber
PE: Plant Extract
TMR: Total Mixed Ration
VFA: Volatile Fatty Acids
ADF: Acid Detergent Fiber
NH3-N: Ammonia Nitrogen
